# Dominant negative biologics normalise the tumour necrosis factor (TNF-α) induced angiogenesis which exploits the *Mycobacterium tuberculosis* dissemination

**DOI:** 10.1186/s12865-023-00576-x

**Published:** 2023-11-30

**Authors:** Kusuma Sai Davuluri, Amit Kumar Singh, Vimal Kumar Yadav, Ajay Vir Singh, Shoor Vir Singh, Devendra Singh Chauhan

**Affiliations:** 1 Department of Microbiology and Molecular Biology, National JALMA Institute for Leprosy and Other Mycobacterial Diseases, Tajganj, Agra India; 2 Department Biotechnology, GLA University, Mathura, India; 3 Department of Animal Experimentation Facility, National JALMA Institute for Leprosy and Other Mycobacterial Diseases, Tajganj, Agra India

## Abstract

**Background:**

Tumor necrosis factor (TNF) is known to promote T cell migration and increase the expression of vascular endothelial growth factor (VEGF) and chemokines. The administration of Xpro-1595, a dominant-negative TNF (DN-TNF) engineered to selectively inactivate soluble TNF (solTNF), has been extensively studied and proven effective in reducing TNF production without suppressing innate immunity during infection. The literature also supports the involvement of glutamic acid-leucine-arginine (ELR+) chemokines and VEGF in angiogenesis and the spread of infections.

**Materials and methods:**

In this study, we administered Xpro-1595 to guinea pigs to selectively inhibit solTNF, aiming to assess its impact on *Mycobacterium tuberculosis (M.tb)* dissemination, bacterial growth attenuation, and immunological responses. We conducted immunohistochemical analyses, immunological assays, and colony enumeration to comprehensively study the effects of Xpro-1595 by comparing with anti-TB drugs treated *M.tb* infected guinea pigs. Throughout the infection and treatment period, we measured the levels of Interleukin-12 subunit alpha (IL-12), Interferon-gamma (IFN-γ), TNF, Tumor growth factor (TGF), and T lymphocytes using ELISA.

**Results:**

Our findings revealed a reduction in *M.tb* dissemination and inflammation without compromising the immune response during Xpro-1595 treatment. Notably, Xpro-1595 therapy effectively regulated the expression of VEGFA and ELR + chemokines, which emerged as key factors contributing to infection dissemination. Furthermore, this treatment influenced the migration of CD4 T cells in the early stages of infection, subsequently leading to a reduced T cell response and controlled proinflammatory signalling, thus mitigating inflammation.

**Conclusion:**

Our study underscores the pivotal role of solTNF in the dissemination of *M.tb* to other organs. This preliminary investigation sheds light on the involvement of solTNF in the mechanisms underlying *M.tb* dissemination, although further in-depth research is warranted to fully elucidate its role in this process.

## Introduction

Tuberculosis (TB) remains a significant global health challenge, despite over a century of active research. It continues to be the leading cause of mortality in humans. This disease is particularly prevalent in underdeveloped regions, claiming a life every 20 s, making it the second most fatal infectious disease after HIV/AIDS [1]. Although TB can affect various organs and tissues, this article focuses on factors contributing to its spread. Extrapulmonary tuberculosis (EPTB) accounts for approximately 20% of all TB cases in immunocompetent individuals and exceeds 50% in those with HIV infection [2]. While pulmonary TB is the most common presentation, dissemination of *M.tb* can lead to miliary tuberculosis [3]. Additionally, EPTB can manifest even without lung involvement, often presenting as pyrexia of unknown origin (PUO) [4]. Several factors are implicated in the dissemination of tuberculosis bacilli, with approximately 15.0% of reactivated TB cases stemming from latent infections [5].

The survival of mycobacteria largely hinges on host immunity. Most *M.tb* infections result in latent TB, characterized by live yet dormant bacilli confined within granulomas. The ability of *M.tb* to circulate in the bloodstream and lymphatic system has been well documented, resulting in TB being detected in nearly every tissue and organ [6]. TNF plays a central role in regulating the host immune response and maintaining the morphology of tuberculous granulomas during the chronic phase of *M.tb* infection. Inhibition of TNF can modulate granulomatous responses by suppressing the production of pro-inflammatory mediators, including chemokines. TNF exists in two forms: transmembrane (tm) TNF and soluble (sol) TNF. Genetic studies in mice have demonstrated that solTNF contributes to disease-related inflammation, while tmTNF [7] is essential for sustaining innate immune function. Overexpression of TNF has been linked to an imbalance in solTNF secretion, resulting in tissue necrosis and cavity formation in mice [8]. solTNF signaling appears associated with persistent inflammation [9, 10], whereas tmTNF signaling plays a role in resolving inflammation and maintaining immunity against bacilli like *M.tb* [10–12]. tmTNF reverse signaling regulates CD4 + and CD8 + T-cell activity and serves a dual function in controlling sTNF bioactivity [13]. The guinea pig serves as a valuable model for human TB, producing primary lesions with central caseous necrosis, mirroring the morphological features of the human disease [14]. Skerry et al. observed that reducing TNF activity during multidrug TB treatment accelerates bacterial clearance [15]. However, agents like Etanercept, which inhibits both solTNF and tmTNF cell signaling, have limitations in TB treatment. Combining TNF suppression with standard therapy enhances bacterial clearance in a mouse model of necrotic TB granulomas [16].

Emerging research suggests that specific inhibition of solTNF through chemical or medication interventions may induce anti-inflammatory responses while preserving innate immunity to infections like *M.tb.* XPro-1595, designed to exclusively inactivate solTNF, has demonstrated utility in animal models of central nervous system (CNS) diseases characterized by elevated TNF production, as well as in mitigating experimental arthritis [17] and endotoxin-induced liver damage without compromising innate immunity to infections [18]. DN-TNF variants act by neutralizing native TNF. In our study, we utilized XPro-1595, a modified form of human soluble TNF featuring mutations Y87H and A145R that abrogate binding to and signaling through TNF receptors TNFR1 and TNFR2. At a lower concentration of 10 ng/ml of Xpro-1595 was found to correlate with a reduction in Nuclear factor- kappa-beta (NF-κB) activation, as well as a decrease in caspase activity. This effect was observed as a result of the exchange of heterotrimeric complexes with native TNF. Importantly, Xpro-1595 selectively interacts with TNF-α but not lymphotoxin-α (TNF-β) [19]. It’s worth noting that XPro-1595 biologic did not block TNF-β, unlike the decoy receptor etanercept. Etanercept acts as a TNFR2 decoy receptor, binding to both TNF and lymphotoxin. Since TNF-β is the closest family member to TNF-α, XPro-1595’s ability to readily interact with solTNF ensures its potential clinical safety, preserving tmTNF’s role in immune function and infection control [20]. The consistent development of primary lesions with central caseous necrosis in the guinea pig model makes it an ideal model for human tuberculosis, closely resembling key morphological aspects of the disease [21].

In our current investigation, we employed XPro-1595 to assess its effectiveness in controlling infection dissemination by specifically targeting solTNF. We observed that *M.tb-*infected animals exhibited elevated levels of ELR + chemokines, VEGFA, and TNF, which may contribute to tuberculosis infection dissemination through the formation of new blood vessels. Furthermore, our research revealed that XPro-1595 treatment effectively restrained the spread of mycobacterial bacilli from the primary site of infection, the lungs, to distant organs. This control was achieved through the modulation of angiogenic factors, including ELR + chemokines and VEGFA.

## Materials and methods

### Experimental ***M.tb*** infection in guinea pigs

*M.tb* H37Rv strain was sourced from the ICMR-NJIL & OMD repository in Agra and cultivated in Middlebrook 7H9 medium (Difco Laboratory, USA), supplemented with 10% albumin dextrose catalase (ADC; Difco Lab.) at 37 °C. The bacterial culture in mid-log phase was quantified and stored in aliquots at -70 °C until further use. Prior to experimentation, the cultures were diluted in sterile normal saline, and the bacterial concentration was adjusted to 1 × 10^6^ CFU/ml. This particular *M.tb* H37Rv strain is a standard choice for animal infection studies in our laboratory [22].

**Fig. 1:**
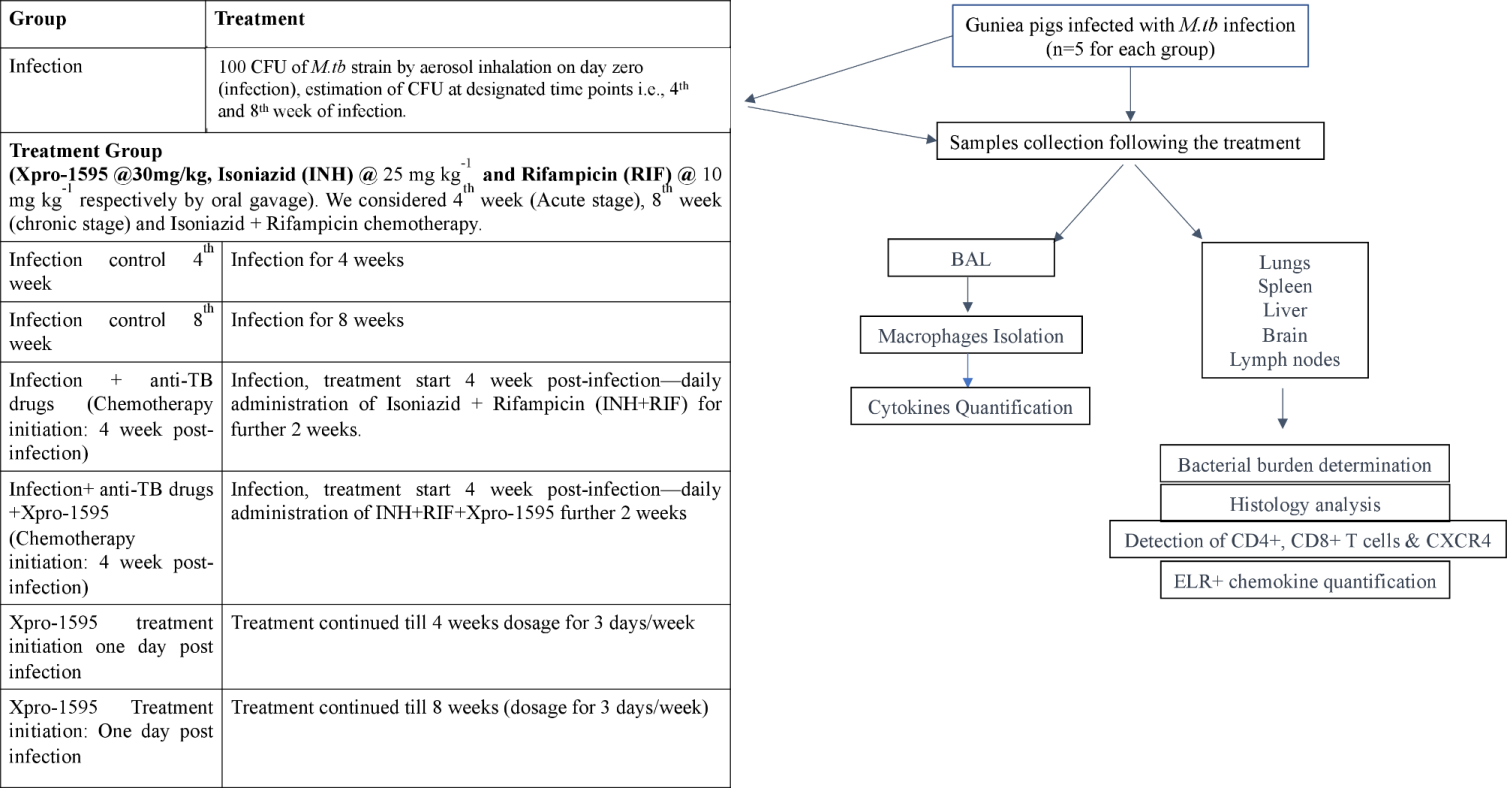
Graphical representation of work flow

Healthy male guinea pigs of the out-bred Hartley strain, weighing approximately 350 g, were procured from Lala Lajpat Rai University of Veterinary and Animal Sciences in Hisar, India. These guinea pigs were housed in a Bio-safety Level-III laboratory under barrier conditions. The infection of guinea pigs with *M.tb* H37Rv was achieved via aerosol exposure, utilizing the Glas-Col whole-body inhalation exposure system (Glas-Col, USA). The system was calibrated to deliver an approximate load of ∼100 CFU to the lungs. Animals were divided randomly into seven groups, with each group comprising five individuals. They were administered 30 mg/kg of Xpro-1595 through slow intraperitoneal infusion following an early-stage *M.tb* infection. Subsequently, first-line drugs, rifampicin (10 mg/kg) and isoniazid (25 mg/kg), were orally administered five days a week for four weeks. After a drug washout period of three days, guinea pigs were humanely euthanized by exposure to isoflurane vapor in a sealed chamber until respiration ceased. The cessation of respiratory and cardiovascular movements was verified by observation for at least 10 min under room air conditions before proceeding with aseptic procedures to access the body cavity. Blood samples were collected via cardiac puncture before the final sacrifice under the influence of anesthesia. Work flow is shown in Fig. 1. This methodology aimed to establish a reliable model of *M.tb* infection in guinea pigs. The purpose was to assess the impact of Xpro-1595 on *M.tb* dissemination and bacterial growth attenuation. Aerosol exposure with a calibrated system ensured consistent infection levels, while drug administration and subsequent sacrifice allowed for the evaluation of treatment efficacy.

### Anti-TNF biologics

The use of DN-TNF proteins was to investigate the role of TNF in *M.tb* infection and the effect of Xpro-1595 on immune responses. The DN-TNF proteins were provided by Xencor Inc. in California. Multiple DN-TNF variants were engineered and produced by them. Bacterial loads and immune responses in all groups were examined at two time points (4th week & 8th week) post-infection. A modified protocol was followed for the *M.tb* solTNF inhibition model [23]. After the initial Xpro-1595 dose of 30 mg/kg administered 2 days pre-infection, dosing continued with 3 doses per week. Infection was allowed to progress for 21 days (acute stage) until granuloma formation. During this drug treatment phase, a modest increase in bacilli count was observed in the DN-TNF therapy group compared to the control groups, which received no therapy until 21 days.

### Colony enumeration assay

Colony Enumeration Assay, conducted during both the 4th and 8th weeks, spanning the early stage and terminal stage of infection, was pivotal in quantifying the bacterial burden within various organs of infected guinea pigs. By assessing the bacterial load at these specific time points, it aimed to provide crucial insights into the effectiveness of Xpro-1595 and anti-TB drugs in curtailing *M.tb* growth across different tissues. Bacterial burden in the lungs, spleen, liver, brain, and lymph nodes of infected guinea pigs was determined during the 8th week post-infection and in Xpro-1595 treated + infected guinea pigs. Organs were weighed and homogenized in PBS. Tenfold serial dilutions of organ homogenates were plated on Middlebrook 7H10 (BD & Co) agar plates containing 10% OADC and incubated at 37 °C for 21 days. Colonies on the plates were enumerated to determine bacterial burden.

### Histology analysis

Hematoxylin and eosin staining allows for the examination of tissue morphology, while fite faracco staining visualizes bacteria, providing insights into the histopathological changes caused by the infection and treatment. Lymph nodes, liver, spleen, brain, and lung tissues collected from sacrificed animals were fixed in 10% buffered formalin and transported out of BSL-III containment. Sections (5 mm) of paraffin-embedded tissues were de-waxed and stained with hematoxylin and eosin (H&E) as described [24], and observations were made under an Olympus BX51 (Olympus, Japan) microscope for histological evaluation. The fite faracco staining method was used to visualize bacteria in the surrounding tissues.

### Isolation of alveolar macrophages (AMs) from guinea pigs

Isolating AMs allows the study of cellular responses in the lungs, a primary site of *M.tb* infection. It helps in evaluating the effects of Xpro-1595 on cytokine production in response to *M.tb* infection. Following anesthesia, the guinea pigs were carefully positioned with the ventral side facing up, and bronchoalveolar lavage (BAL) fluid was collected as described previously [25]. Briefly, the abdominal and thoracic cavities were gently opened to expose the organs without causing damage. Subsequently, the lungs were flushed with ice-cold PBS until they appeared blanched as described previously. Once isolated, BAL cells were cultured in vitro and adherent alveolar macrophages were isolated and further cultured for experimentation. The harvested cells were resuspended in an appropriate culture medium, and their viable cell concentration was adjusted. The cells were then seeded in culture vessels and incubated under controlled conditions at 37 °C with 5% CO_2_. After 24 h, the culture medium was replenished, and the cells were ready for use.

### Evaluation of inflammatory cytokines in bronchoalveolar lavage (BAL) samples

Measuring cytokine levels in BAL samples aims to understand the local immune response in the lungs. This helped in assessing the impact of Xpro-1595 on cytokine production and its role in modulating inflammation during *M.tb* infection. The expression of TNF, IFN-γ, and IL-12 were determined through ELISA by separating the macrophages from the BAL samples. Anti-TGFB1, Anti-IL-12, and Anti-IFNGR2 produced in rabbits (Sigma Aldrich, USA) were used to perform ELISA. 75 μl of primary antibody (1:10000 dilution) was added to each well and incubated at 4 °C overnight. The plate was washed three times for five minutes each. 300 μl of blocking buffer was added to each well. The plate was covered and incubated at room temperature for 2 h. Blocker was removed, and a sample of 75 μl was added to each well. The plate was covered and incubated for 2 h at room temperature. After that, the plate was washed three times for 5 min each. A 1:1000 ratio of Enzyme conjugate HRP- anti-goat IgG secondary antibody (1:10000 dilution) was added to each well and incubated for 1 h at room temperature following the washing step. OPD solution was added to each well and developed at room temperature for 30 min; later to stop the reaction, 50 μl of 70% H_2_SO_4_ was added. Quantification was performed by calculating the concentration/ml through the OD values at 490 nm.

### Quantification assay based on multiple primer sets

Quantifying the expression of specific genes related to immune responses might provide molecular insights into the effects of Xpro-1595 on the expression of ELR + chemokines, which are crucial in the immune response against *M.tb*. Lungs were ground in liquid nitrogen and transferred to a pre-chilled 2ml microcentrifuge tube. Isolation of RNA was done using the Fermantas Kit as per the manufacturer’s instructions, followed by quantification on Nanodrop 2000c (Implen, Germany), and RNA quality was checked using agarose (2%) gel electrophoresis. RT-PCR was performed for ELR + chemokines using primers designed with the primer3 software (Table-1).

**Table 1:** Primers for ELR + chemokine ligands for guinea pig

Gene	Primer sequence	Annealing temperature	Product size
CXCL-1	F- CACCCCAAGAACATCCAGAG	60^0^ C	157 bp
	R- TGCTTCCTTTCAGCATCCTC		
CXCR-2	F-CAAAACAAGGCCATCTTGT	55^0^ C	247 bp
	R-CATCTACCATGGAGCCACT		
CXCL-6	F-GCCTGCAAGTGACTGTCTA	59^0^ C	74 bp
	R-CTGCTTCCCATTCTTCAAG		
CXCL-7	F ACCCAGTGTGAAGTCCTTGG	59^0^ C	202 bp
	R-TGATGCCCAGTAACCTAGC		
CXCL-8	F-GACAAAGTTGGTCTCGC	55^0^ C	150 bp
	R-GCAGAGCTGTCGATTGC		
CCL-2	F-TGCCAAACTGGACCAGAGAA	55^0^ C	72 bp
	R-CGAATGTTCAAAGGCTTTGAAGT		
CCL-11	F-CCCTGCCATGTGTAGGAAT	57^0^ C	150 bp
	R-TCACAACCTGGCTTCACAG		
CCL-16	F-CCTGTGTCTGTCCCCAAGT	55^0^ C	249 bp
	R-ATCCCAACACCACTTTCTC		
VEGFA	F-GGCCCATCGAGATGCTAGTG	60^0^ C	276 bp
	R- GCCCACAGGGATTTTCTTGC		

### Detection of antigens within tissue sections

Immuno-histochemical staining allows the visualization of specific antigens, such as CD4 and CXCR-4, within tissue sections. This helped in assessing the distribution and presence of immune cells and receptors, shedding light on the immune response in the infected tissues. De-waxed tissue sections were subjected to immune-histochemical staining using streptavidin peroxidase and fluorochrome-conjugated antibodies following the manufacturer’s protocol with slight modification (Daako Flex®, Denmark) [26]. Anti-CD4 (produced in rabbit) were purchased from Vector Laboratories (USA), while PE-conjugated CXCR-4 antibody (Elab Bioscience, China) were used for immuno-fluorescent-staining with DAPI as a counterstain [27]. Briefly, tissue sections were incubated with Anti-CD4, and CXCR-4 antibodies for one hour. Excess antibody was removed by washing with phosphate buffer saline (PBS) at room temperature for 30 min and incubated with secondary detection antibody rabbit anti-mouse conjugated to HRP. End product visualization was achieved using 3’,3-diaminobenzidine tetrahydrochloride (DAB) (Sigma, USA)/H_2_O_2_ as the chromogen.

### Statistical analysis

**Fig. 2:**
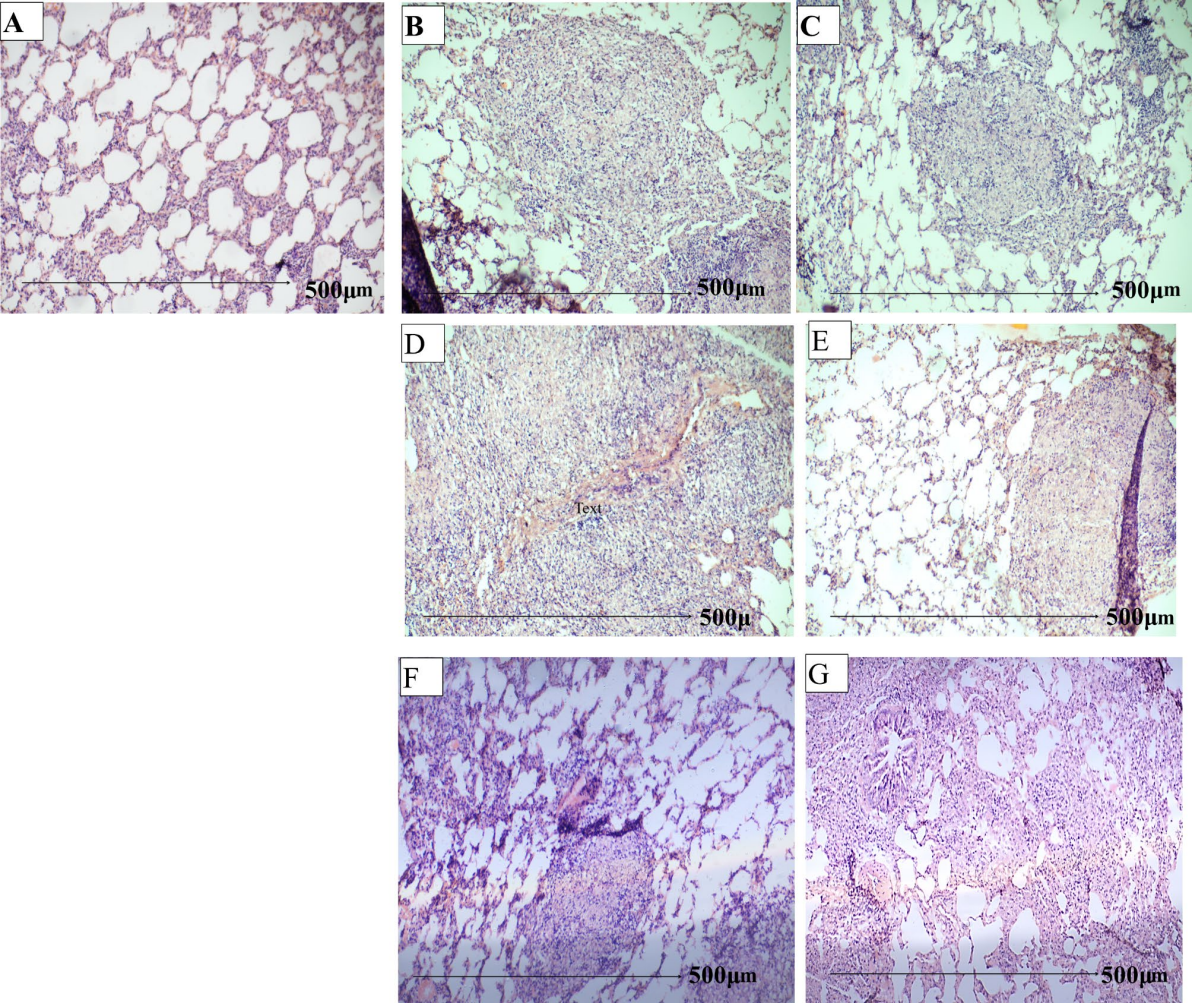
The images shown are representative of sections obtained from 5 animals per group. (**A**). Histology of lung in a healthy guinea pig. (**B**,**C**). *M.tb* infected and *M.tb* infected Xpro-treated guinea pigs 4th week post-infection. (**D**, **E**). *M.tb* infected, and *M.tb* infected Xpro-treated guinea pigs, 8th week post-infection. Solid granulomas exhibited caseous necrotic lesions with no cellular regions in the *M.tb* infected guinea pigs. Granulomas in Xpro-treated guinea pigs showed reduced necrosis and open-air spaces. (**F**) (**G**). Lung pathology in animals receiving anti-TB drugs (Isoniazid (H) and rifampicin (R)) adjunctive Xpro-1595 (H plus Xpro-1595) appeared to resolve with more open-air spaces than those receiving standard treatment (RH alone) respectively. Experiments were performed on triplicate sections collected from n = 5 guinea pigs in each group. All data in each figure is from one biological repeat. *M.tb* output was expressed as mean log _10_ CFU/ml ± SE (n = 5). One-way ANOVA test was used to compare the differences between treated and untreated cultures with statistical significance indicated as *** (p < 0.001)

Statistical significance was evaluated using GraphPad Prism 9. Fractal analysis of granulomas and CXCR4 expression (Fig-1) was measured using Magvision software, and statistical significance was calculated using the area covered in the same panel 500μ, with a p-value < 0.01 considered significant. For qRT-PCR (Fig-2), statistical significance was calculated using the two-way ANOVA test on ΔΔCt relative transformation fold changes, with a p-value < 0.01 considered significant.

## Results

### Decreased pathogenesis in Xpro-1595 treated guinea pigs

At week 4, examination of guinea pig lungs revealed the presence of conspicuous tubercles in our previous study [28, 29] and histological analysis unveiled the presence of poorly formed granulomas characterized by epithelioid histiocytes with a sparse lymphocyte and plasma cell population, along with speckled necrosis (Fig. 2B). Treatment with adjunctive Xpro-1595 demonstrated a reduction in TB-associated pathology as shown in 2G. Hematoxylin and eosin staining (10X) of lung tissues highlighted that granulomas in Xpro-1595 treated guinea pigs displayed decreased necrosis, suggesting a potential reduction in cellular and humoral mediators responsible for tissue damage (Fig. 2). Different scoring parameters for histopathology at the 4th week and 8th week are presented in Table-2.

**Table 2:** Scoring parameters for histopathology at 4th and 8th week post infection *M.tb* infected and *M.tb* infected Xpro-1595 treated

Parameters	Different therapeutic groups of animals					
	*M.tb* infection 4th week	*M.tb* infection + Xpro-1595 treated 4th week	*M.tb* infection 8th week	*M.tb* infection + Xpro1595 treated 8th week	*M.tb* infection + DOTS	*M.tb* infection + DOTS + Xpro1595 treated 8th week
Infiltration factor (% mean)	65%	40%	40%	45%	57.5%	35
No. of lesions (mean)	7.3	3.5	6	4.5	3	3
Granuloma/non granuloma	++/+	++/+	++/++	++/++	++/+	++/+
Necrosis	+++	++	++	+	++	+
Cell population	Mø, Ly	Mø, Ly, Gaint cells	Mø, Ly	Mø, Ly	Mø, Ly	Mø, Ly
AFB	2+	2+	1+			

Mø-Macrophages, Ly-Lysosomes, + : Present, - : Absent

### DN-TNF therapy reduced dissemination during the 8th week of ***M.tb*** infection

**Fig. 3:**
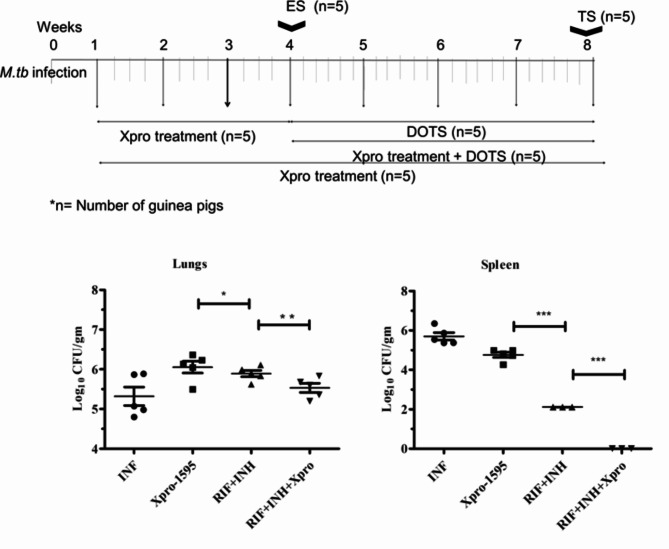
Graph showing the data obtained from 5 animals per group. All data in each figure is from one biological repeat. CFU counts at an 8th -week time point in guinea pig lung tissue. The graph shows a significant decrease in CFU count in treated groups. Twenty-one days of drug therapy with Xpro-1595 showed reduced viable bacilli in the lungs and spleen. Dissemination of bacilli was less in the spleen in Xpro-1595 treated guinea pigs when compared with *M.tb* infected guinea pigs without treatment (p < 0.001). Standard deviation was > 0.1

Treatment with Xpro-1595 for four weeks before initiating anti-TB drug therapy did not significantly impact mycobacterial survival in the lungs and spleen when compared to infected, non-treated animals. After four weeks of infection, the administration of the anti-TB drug combination (10 mg/kg rifampicin + 25 mg/kg isoniazid) five times a week for four weeks demonstrated potent bactericidal activity (Fig. 3).

### Detection of bacilli in lymph nodes, liver, and brain

**Fig. 4:**
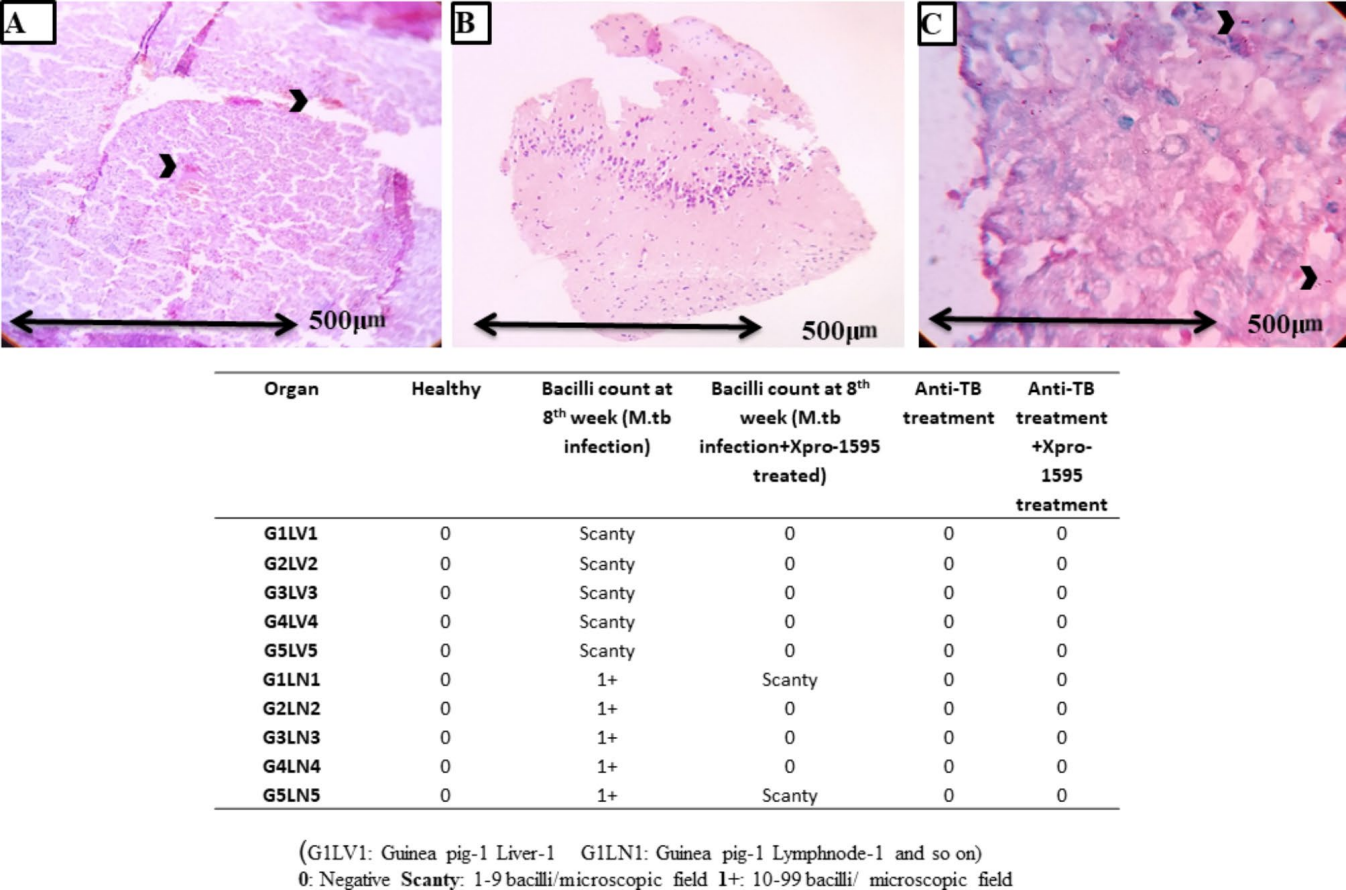
Liver, lymph nodes, and brain samples were separated to examine the bacterial presence, and total tissue samples were collected from n = 5 number of guinea pigs from each group, examined with triplicates to avoid false negative/false-positive results in the overall analysis. Each figure displays data from a single biological replicate. (**A,B**). Showing the AFB + bacilli at 100X magnification during 8th week *M.tb* infection in guinea pig’s lymph node. (**C**). Showing the bacilli in liver tissue at 100X magnification during the 8th week *M.tb* infection

During the 4th week of *M.tb* infection, bacilli had not disseminated from the lungs to the liver, with no microscopically visible lesions in the liver and lymph nodes. Bacterial counts in lymph nodes and the liver were negligible in XPro-1595 treated Guinea pigs (data not shown). Conversely, an increase in bacterial count was observed in the liver and lymph nodes of the *M.tb* infected control group. Notably, bacterial infection was not detected in the liver and lymph nodes of XPro-treated guinea pigs. Furthermore, a lower bacterial count was observed in the spleen compared to the control group. Interestingly, no bacilli were found in the brain region for both groups. The fite-faracco staining method confirmed the absence of bacilli in liver and lymph node samples of XPro-1595 treated Guinea pigs (Fig. 4).

### Selective inhibition of solTNF by XPro-1595 alters TNF levels

**Fig. 5:**
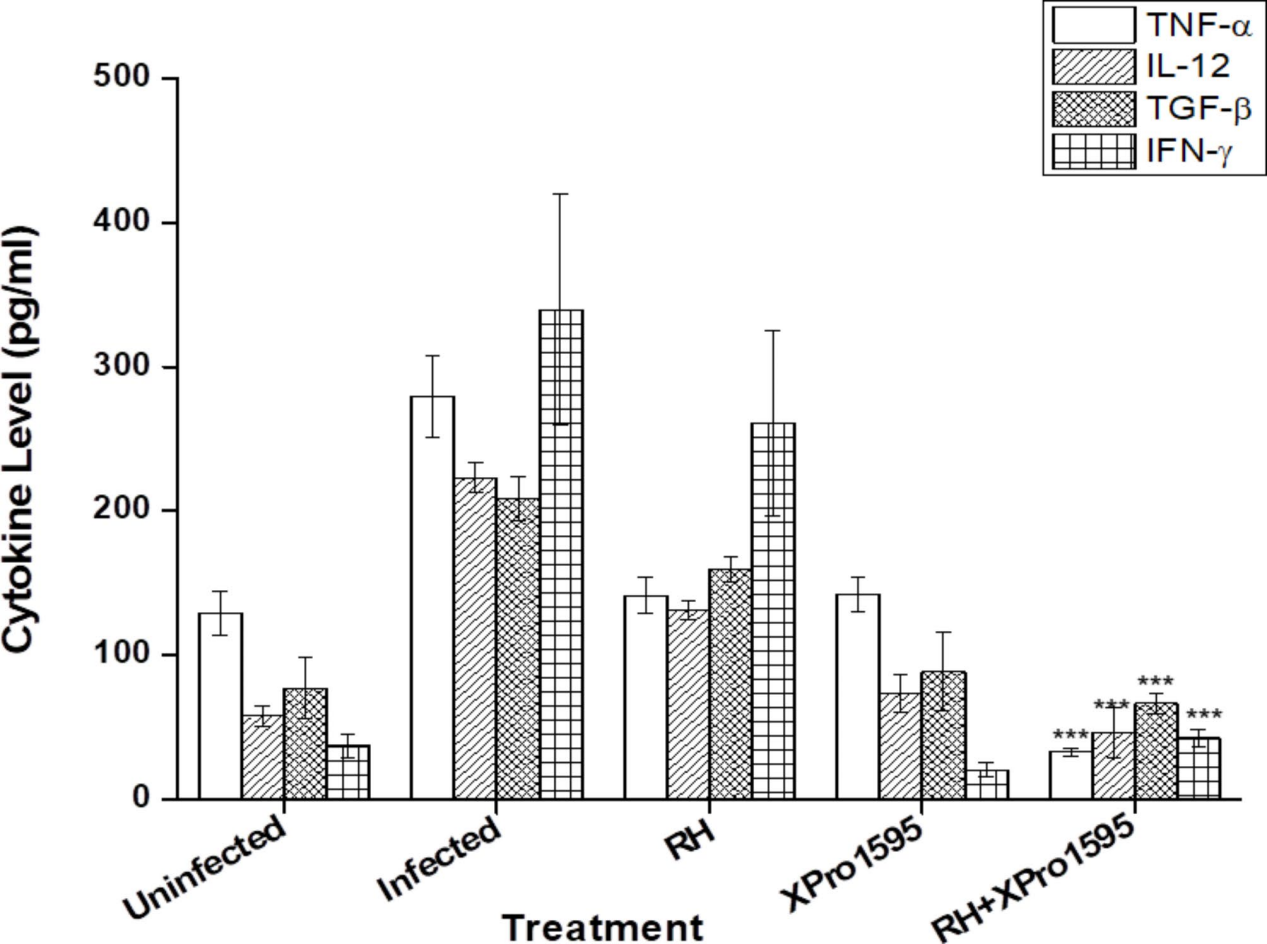
Cytokine activity was measured in BAL fluid by ELISA. Levels for guinea pigs non-infected or infected receiving no treatment (Untreated), Standard TB treatment (RH), or with adjunctive Xpro-1595 (RH + Xpro-1595) were measured 28 days after the start of treatment. TNF activity decreased with TB treatment and was abrogated by the administration of adjunctive Xpro-1595. Samples were collected from n = 5 number of guinea pigs from each group, examined with triplicates to avoid false negative/false-positive results in the overall analysis. Results were plotted based on mean concentrations. *M.tb* infected, INH + RIF treatment groups were compared with Xpro-1595 + INH + RIF treatment group *** *p* < 0.005

We examined the levels of three pro-inflammatory cytokines: TNF-α, IL-12, IFN-γ, and the anti-inflammatory cytokine TGF-β through ELISA. As expected, TB infection led to an increase in TNF activity compared to uninfected guinea pigs. Treatment with either anti-TB drugs or Xpro-1595 resulted in a significant reduction in TNF activity when compared to the infected group. The increased TNF activity was further diminished by the administration of adjunctive Xpro-1595 (p < 0.01). Additionally, the adjunctive therapy of Xpro-1595 with anti-TB drugs enhanced IFN-γ cytokine secretion, restoring it to levels comparable to the control group. (Fig. 5).

### Reduction of elevated ELR + chemokines in XPro-1595-treated ***M.tb***-infected guinea pigs

**Fig. 6:**
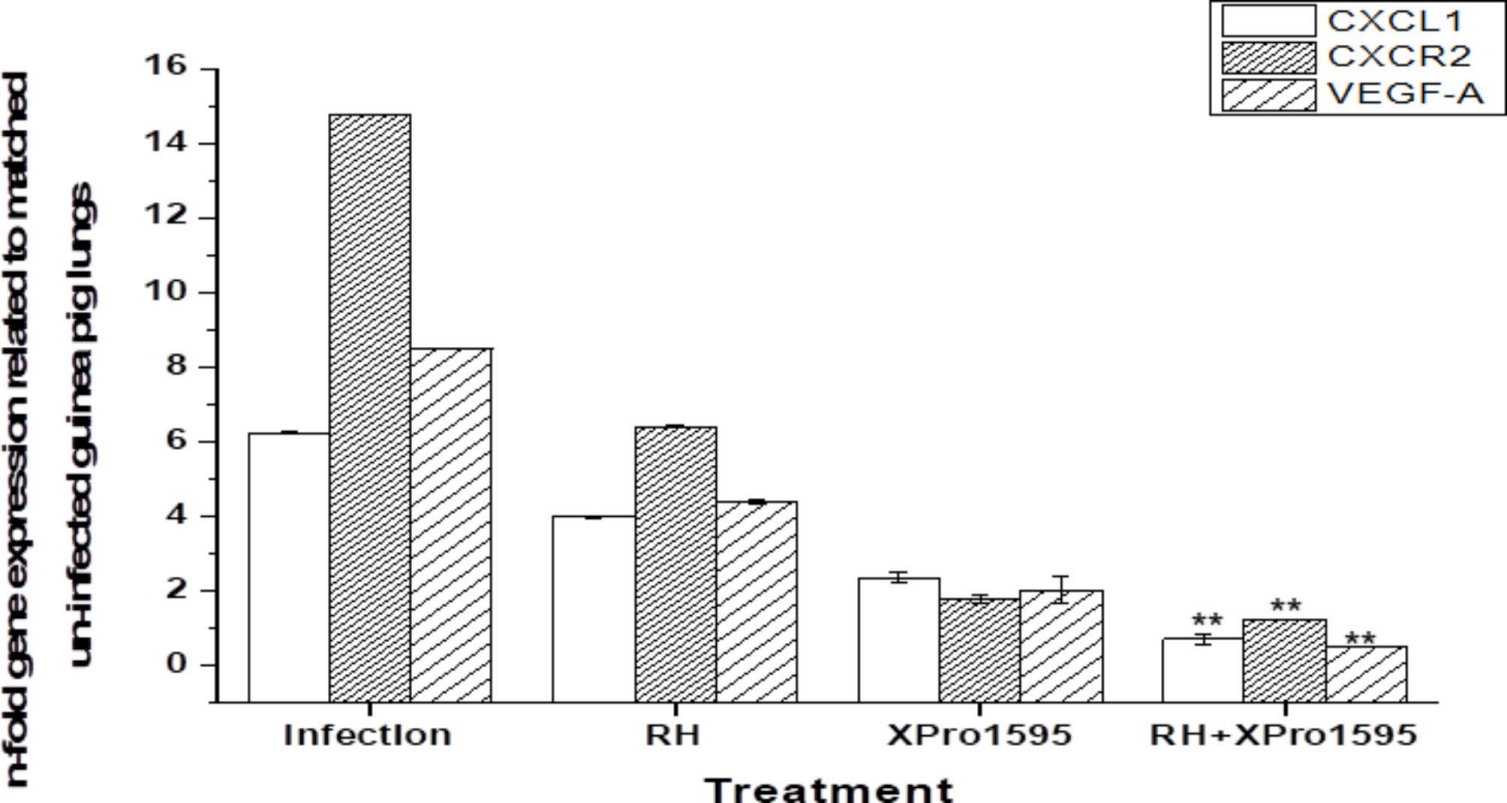
Expression of CXCL1, CXCR2, and VEGF-A in *M.tb* infected guinea pig lungs treated with anti-TB drugs rifampicin(R) and isoniazid (H). Xpro-1595 as an adjunct to anti-TB drugs as determined by qRT-PCR performed in triplicates. qRT-PCR data are expressed as mean +/- standard error of the mean **p < 0.05, n = 5, respectively

Treatment with Xpro-1595 significantly reduced the expression of the CXCL1/CXCR2 axis. Furthermore, treatment with anti-TB drugs attenuated the mRNA levels of CXCL1/CXCR2, which were further reduced in guinea pigs treated with the combination of anti-TB drugs and Xpro-1595. Similar results were observed for VEGF-A, with the combination of Xpro-1595 and anti-TB drugs significantly reducing VEGF-A levels compared to anti-TB drugs alone, effectively lowering angiogenic factor levels to those of the control group (Fig. 6).

### XPro-1595 treatment shows a potential reduction in CD4 + T cell count without observable impact on bacterial severity

**Fig. 7:**
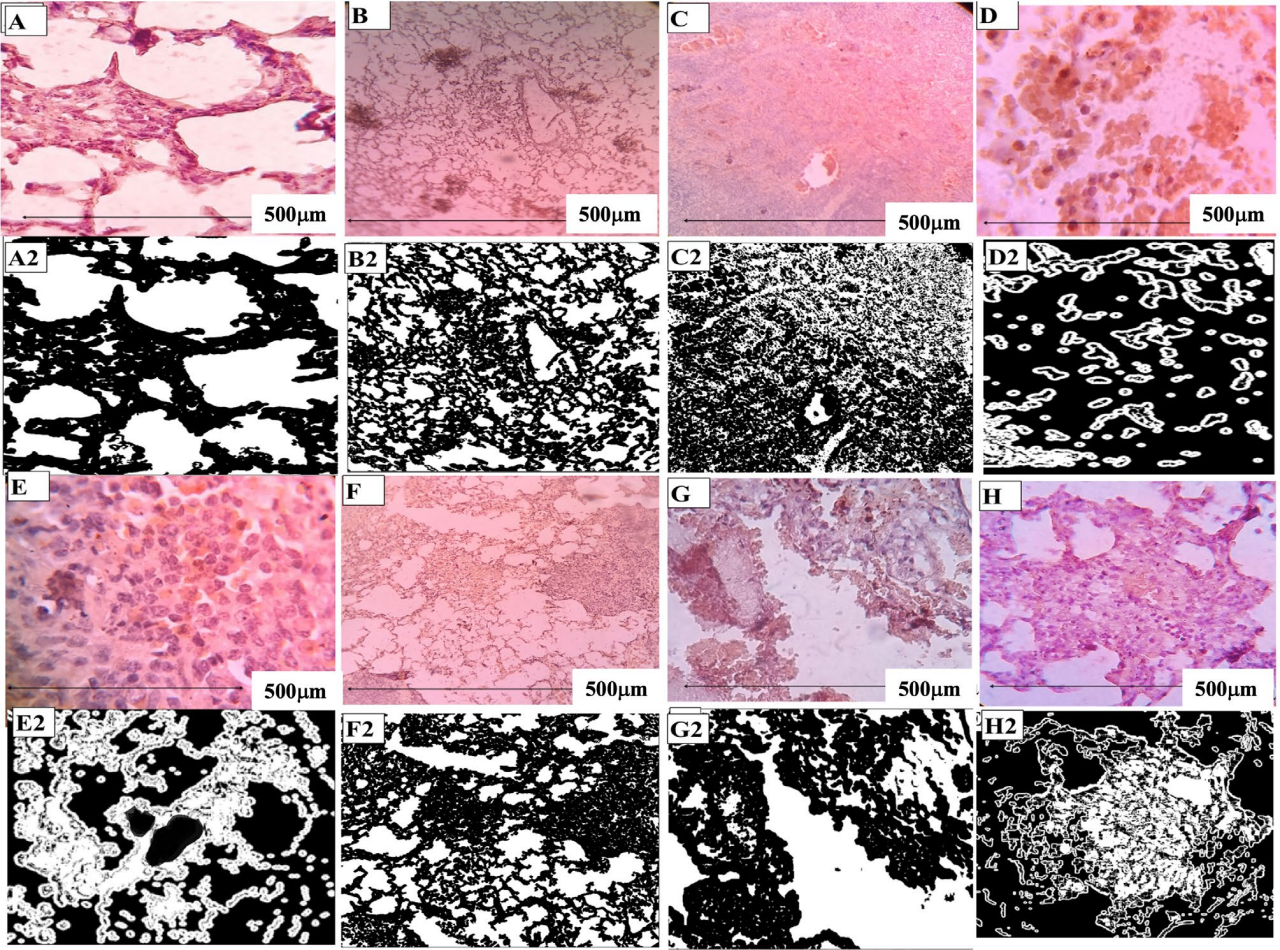
(**A**). CD4 + T cell expression in healthy guinea pig 7B,7 C. CD4 + T cell expression was significantly elevated in *M.tb* infected guinea pigs during the 4th week (cell count mean value/unit area = 188.54) as compared to that in *M.tb* infected Xpro-1595 treated guinea pigs (cell count represent value/unit area- 160.63) respectively; the results were obtained using triplicate sections of tissue followed by analysis using IHC tool plugin in ImageJ software. (**D** and **E**). CD4 + T cell expression was significantly reduced in both *M.tb* infected guinea pigs, and *M.tb* infected Xpro-1595 treated guinea pigs during the 8th week compared to that in the 4th week; the results were obtained using IHC tool plugin in ImageJ software. (**F**). The Xpro-1595 treated group alone (**G, H**). During the treatment with RH + Xpro-1595, we found a reduced CD4 + T cell count compared with RH treatment group providing the evidence of an early diminish in bacteria after the chemotherapy. **A2, B2, C2, D2, E2, F2, G2** and **H2** are staining detection images identified by ImageJ that distinguishes DAB and HE stain. The experiments were conducted in triplicate on tissues collected from five guinea pigs in each group. Each figure displays data from a single biological replicate

During the early stages of TB infection, we observed significant changes in the migration of CD4 + T cells in *M.tb*-infected Xpro-1595 treated guinea pigs and *M.tb*-infected guinea pigs. This might indicate that excessive migration of T cells might induce inflammation without significantly affecting bacterial severity. By the 8th week, adjunctive treatment of Xpro-1595 with anti-TB drugs led to reduced CD4 + T cells compared to anti-TB drug treatment alone, suggesting early restriction of bacterial burden. Our analysis showed a positive association between CD4 + T cell immune staining at the infection site, bacterial load, the number of lesions (as shown in Table-1), and the number of affected cells in *M.tb*-infected guinea pigs after the 4th week of post-infection (Fig. 7).

### solTNF-α dependent expression of CXCR4 in ***M.tb*** infected guinea pigs

**Fig. 8:**
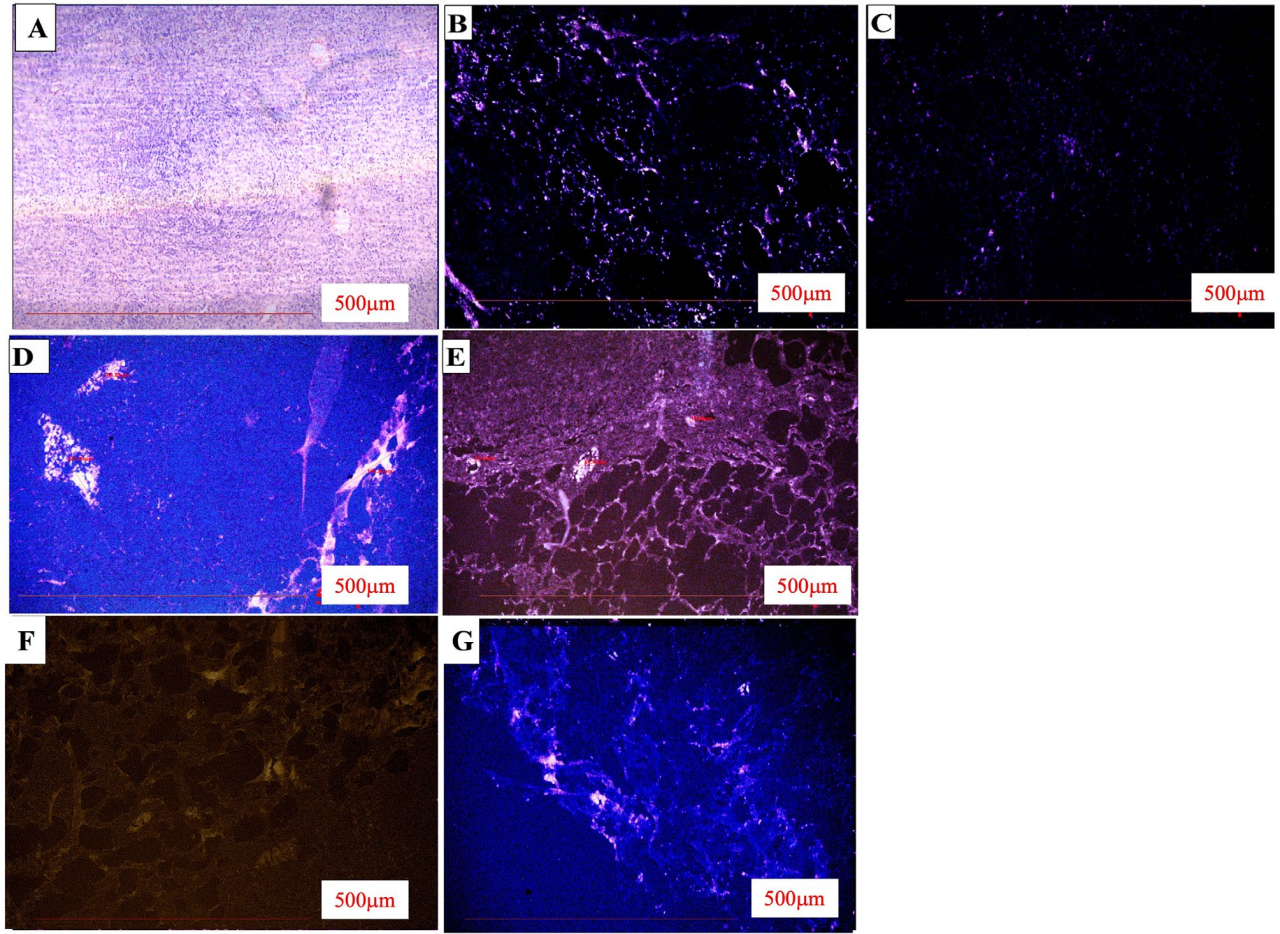
Variable expression of CXCR4 was observed in triplicate sections of tissues collected from the experimental guinea pigs. *M.tb* infected Xpro-1595 treated guinea pig’s lung tissue across the spectrum. **A.** Control **B.***M.tb* infected 4th week. **C.** Xpro-1595 treated 4th week **D.***M.tb* infected 8th week **E.** Xpro-1595 treated 8th week **F.** RH treated guinea pig lung tissue sections **G.** RH + Xpro-1595 treated. Experiments on expression of CXCR4 was performed in triplicates on tissues collected from n = 5 Guinea pigs from each group. All data in each figure is from one biological repeat

We observed that the chemokine receptor CXCR4 was significantly expressed in the lungs of *M.tb*-infected guinea pigs during 4th week and 8th week (Fig. 8B and D) respectively when compared with the uninfected guinea pigs (Fig. 8A). Treatment with Xpro-1595 reduced CXCR4 expression significantly as shown in Fig. 8C. Additionally, treatment with anti-TB drugs attenuated the level of CXCR4, which was further reduced in guinea pigs treated with the combination of anti-TB drugs and Xpro-1595 as shown in Fig. 8E F respectively when compared with guinea pigs *M.tb* infected controls (Fig. 8D). We detected the fewer signals of CXCR4 expression in the solTNF inhibited group showing the regulation of chemokine receptor expression through the solTNF factor (Fig. 8G), (Table-3).

**Table 3:** IHC stained lung tissue showing CD4 + T cell expression/unit area in various groups

Parameters	Different therapeutic groups of animals						
	*M.tb* infection 4th week	*M.tb* infection + Xpro-1595 treated 4th week	*M.tb* infection 8th week	*M.tb* infection + Xpro1595 treated 8th week	*M.tb* infection + DOTS	*M.tb* infection + DOTS + Xpro1595 treatment	Healthy
No. of cells/Unit area (mean)	214.31	175.8	162.52	176.73	139.28	103.43	77.83
P-Value	P**		No significance		P**		

## Discussion

In late-stage TB infection, a delicate balance between proinflammatory and anti-inflammatory immune responses becomes critical. The precise involvement of T cells as protective elements in both the early and late stages of infection remains inadequately understood. It is essential to maintain a balance between anti-inflammatory and pro-inflammatory cytokines during the chronic stage of infection. Cytokines, often expressed in soluble forms by one cell type and acting on another, induce changes in the target cell’s function. Pharmacological inhibition of soluble TNF may demonstrate anti-inflammatory properties while preserving innate immunity [30, 31]. Few studies highlight the intricate regulatory mechanisms involving transmembrane TNF and its receptors, TNFR1 and TNFR2, in the context of mycobacterial infections and pleurisy-induced inflammation. They underscore the pivotal role of transmembrane TNF in modulating myeloid-derived suppressor cell activity and emphasize the importance of TNFR2 in mitigating excessive inflammatory responses. The findings contribute significantly to our understanding of the immunopathology associated with mycobacterial infections, offering potential insights for therapeutic interventions aimed at controlling inflammation and enhancing host defence mechanisms in such contexts [32–34].

DN-TNF therapy effectively reduces inflammation, resulting in improved lung conditions with fewer pathological alterations. By the 8th week, well-formed caseous granulomas are observed in untreated infected animals. Treatment with XPro-1595 reduces necrotizing lesions and improves lung functionality. Conversely, histological evaluations of lung samples from animals treated with the standard anti-TB drugs isoniazid and rifampicin reveal significantly reduced inflammation and improved lung function, with smaller granulomas primarily confined to the lung periphery. Importantly, treatment with XPro-1595, either alone or as an adjunct to anti-TB drugs, does not adversely affect mycobacterial survival in the lungs compared to the infected control or anti-TB treated guinea pig groups.

Host immunity demonstrates its capacity to provide protection against *M.tb* infection during the 8th week, but it struggles to control dissemination to peripheral sites such as the liver and lymph nodes. IL-12, another pro-inflammatory cytokine, has also been implicated in angiogenesis in TB infection [35]. Hence, the effect of DN-TNF therapy on IL-12 was also assessed. The IL-12 levels followed a similar pattern to TNF (I > RH > XPro-1595 > XPro-1595 + RH). IFN-γ, another pro-inflammatory cytokine, is believed to be induced by TNF via IL-1 [36]. IFN-γ, in turn, stimulates the release of TNF, perpetuating a cycle of chronic inflammation [37].

Chemokines play a crucial role in directing immune cells to inflammatory sites, potentially leading to unregulated angiogenesis that impacts disease severity. Therefore, studying the regulatory mechanisms controlling the production of angiogenesis-related chemokines is essential. The majority of previous studies have emphasized the clinical characteristics of CXCR4 positive cells, which are rare in healthy donors and absent in cord blood. Numerous host factors regulated by TNF are responsible for TB infection dissemination [38]. Our study suggests that DN-TNF agents can balance the need to reduce inflammation while maintaining resistance to infection and microbial diseases [39]. The dissemination of mycobacterial infections often occurs due to excessive or deficient immune responses. The discovery of TNF inhibitor biologics that specifically inhibit soluble TNF while sparing transmembrane TNF is highly valuable [40]. Our study explored the role of soluble TNF in controlling *M.tb* infection dissemination while sustaining both acute and chronic bacterial growth inhibition and T-cell migration to the infection site. Two possible outcomes are systemic dissemination or bacterial containment [41]. The activity of TNF induces a cytokine storm, supporting the release of chemokines that recruit various immune cells [42].

Anti-TNF agents, such as adalimumab, have been shown to cause disseminated TB in non-human primates [43]. Alterations in macrophage function and a reduction in CD8 + CCR7 + CD45RA + effector memory T-cells are likely mechanisms involved in promoting TB disease during TNF neutralization treatment [44], leading to dissemination. We found that soluble TNF is involved in the migration of CD4 + cells during infection and the synthesis of chemokines. We observed an increase in the expression of ELR + chemokines from the 4th to the 8th week post *M.tb* infection, controlled by XPro-1595 treatment.

Our observations support the idea that early TB dissemination in *M.tb*-infected guinea pigs is correlated with the early initiation of anti-mycobacterial immunity. Excessive migration of immune cells to the infection site induces inflammation but might provide access for bacteria to disseminate through lymphatics. Treatment with XPro-1595 inhibits *M.tb* migration from the lungs to the spleen, liver, and lymph nodes, reducing dissemination and inflammation while maintaining normal cell morphology. VEGFA and ELR + chemokines appear to be induced by TNF and play a role in infection dissemination. These factors are also involved in the migration of CD4 T cells in the early stage of infection.

In conclusion, our findings suggest that specific inhibition of soluble TNF by XPro-1595 can control the increased expression of TNF-induced *M.tb* dissemination. The adjunctive use of TNF inhibitors during TB treatment may be beneficial. We further investigated the effect of soluble TNF inhibition on angiogenic factors in *M.tb*-infected guinea pigs. This study highlights the potential of TNF inhibitor biologics to modulate the immune response and reduce TB-associated pathology.

## Conclusion

In the intricate world of tuberculosis research, a critical interplay unfolds between proinflammatory and anti-inflammatory responses, where T cells play a central role. Understanding this interplay is essential, particularly in the chronic stages of TB. Researchers have long sought to strike a balance between these immune responses during late-stage TB infection. This quest led to the exploration of cytokines, molecular messengers that influence immune responses. solTNF a key cytokine, emerged as a potential therapeutic target. tmTNF controls myeloid-derived suppressor cells, while TNFR2 helps dampen excessive inflammation. These findings offer promise for therapies to control inflammation and enhance host defence. Enter DN-TNF therapy, which acts as antagonist for solTNF might effectively reduces inflammation and improves lung function without compromising the body’s ability to combat TB. Our study delved into the world of chemokines and cytokines which are critical agents directing immune cells to infection sites but also potentially causing unregulated angiogenesis. We understood the importance of regulating angiogenesis-related chemokines, and DN-TNF therapy aimed to strike that delicate balance. Despite improved lung conditions, TB often spreads to peripheral sites like the liver and lymph nodes. Pro-inflammatory cytokines like IL-12 and IFN-γ might contribute to this process, forming a complex loop of chronic inflammation. The study also delves into chemokines, crucial for directing immune cells to infection sites but potentially causing unregulated angiogenesis. Understanding the regulation of angiogenesis-related chemokines is key, and DN-TNF therapy aims to strike a balance. In summary, research efforts seek to balance inflammation and immunity in the TB battle. The development of XPro-1595, a specific soluble TNF inhibitor, offers hope for controlling TB-related pathology and modulating the immune response.

## Data Availability

The datasets used and analysed in current research are provided on reasonable request which are available from corresponding author.
